# Microwave Irradiation as a Powerful Tool for Isolating Isoflavones from Soybean Flour

**DOI:** 10.3390/molecules29194685

**Published:** 2024-10-02

**Authors:** Sanja Đurović, Bogdan Nikolić, Boris Pisinov, Dušan Mijin, Zorica Knežević-Jugović

**Affiliations:** 1 Institute for Plant Protection and Environment, Teodora Drajzera 9, 11040 Belgrade, Serbia; stojakovicsm@yahoo.com (S.Đ.); bogdannik@mail2world.com (B.N.); boriss752002@yahoo.com (B.P.); 2 Department of Organic Chemistry, Faculty of Technology and Metallurgy, University of Belgrade, Karnegijeva 4, 11000 Belgrade, Serbia; kavur@tmf.bg.ac.rs; 3 Department of Biotechnology and Biochemical Engineering, Faculty of Technology and Metallurgy, University of Belgrade, Karnegijeva 4, 11000 Belgrade, Serbia

**Keywords:** microwave irradiation, isoflavones, soybean flour, antioxidants, extraction technique

## Abstract

The use of microwave irradiation energy for isolating bioactive compounds from plant materials has gained popularity due to its ability to penetrate cells and facilitate extraction of intracellular materials, with the added benefits of minimal or no use of organic solvents. This is particularly significant due to the possibility of using extracts in the food and pharmaceutical industries. The aim of this work is to examine the effect of microwave irradiation on the extraction of three of the most important isoflavones from soybean flour, glycitin, genistin, and daidzin, as well as their aglycones, glycitein, genistein, and daidzein. By varying the extraction time, temperature, and microwave power, we have established the optimal parameters (irradiation power of 75 W for 5 min) for the most efficient extraction of individual isoflavones. Compared to conventional maceration and ultrasound-assisted extraction, the total phenol content of the extracts increased from 3.66 to 9.16 mg GAE/g dw and from 4.67 to 9.16 mg GAE/g dw, respectively. The total flavonoid content increased from 0.38 to 0.83 mg CE/g dw and from 0.48 to 0.83 mg CE/g dw, and the antioxidant activity increased from 96.54 to 185.04 µmol TE/g dw and from 158.57 to 185.04 µmol TE/g dw, but also from 21.97 to 37.16 µmol Fe^2+^/g dw and from 30.13 to 37.16 µmol Fe^2+^/g dw. The positive correlation between microwave extraction and increased levels of total phenols, flavonoids, and antioxidant activity demonstrates the method’s effectiveness in producing bioactive compounds. Considering the growing recognition of glycitein’s potential role in medical and pharmaceutical applications, microwave-assisted extraction under optimized conditions has proven highly efficient.

## 1. Introduction

**Figure 1:**
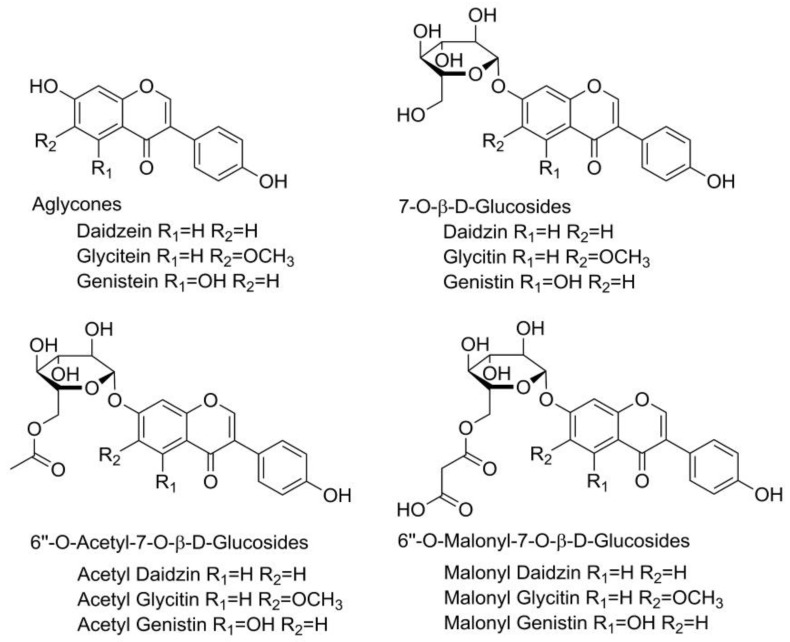
Structural formula of twelve soybean isoflavones.

Polyphenols are naturally occurring antioxidant compounds found in plants and foods of plant origin, receiving tremendous attention from nutritionists, food scientists, and consumers due to their roles in human health [1,2]. Isoflavones are a subclass of polyphenolic compounds found mainly in soybeans as well as other legumes [3,4]. These compounds are produced through phenylpropanoid pathways in the plant as a part of its secondary metabolism and play a crucial role in the plant’s survival. They act as hydrogen and free radical acceptors, protecting the plant from oxidative stress caused by adverse environmental conditions such as harsh weather conditions, the presence of microorganisms, and mechanical damage. They also have functions in enzyme activity, photosynthesis, nutrient assimilation, protein synthesis, and cell signaling [5]. In plant materials, isoflavones can be present in their free form (aglycones), but are more often bound to a sugar component (glucosides, their 6″-O-malonyl-β-glucosides (6OMalGlc); and their 6″-O-acetyl-β-glucosides (6OAcGlc) conjugates). The aglycone form of isoflavones is responsible for their biological activity. Aglycones are found in larger quantities in fermented food; they are more soluble in lipids and therefore can easily pass through the intestines, resulting in increased bioavailability [6]. The glucosidic form can be converted to its aglycone form by hydrolysis, which begins in the mouth during food intake and continues in the stomach and intestines. The liver and both small and large intestines also contain intestinal hydrolytic enzymes and microbial glucosidases that can break down glucosides [7]. Soybean, as a very important industrial plant, is extremely rich in isoflavones and aglycones, including genistein, glycitein, and daidzein, as well as their β-glucosides, acetyl glucosides, and malonyl glucosides (Figure 1).

For centuries, it has been a part of people’s everyday diets, particularly in Asia. However, in the last few decades, soy and soy products have become increasingly important in the worldwide diet because of their beneficial nutritional content. Numerous studies demonstrated that regular daily consumption of soy isoflavones can provide several health benefits and even help prevent certain diseases. Soy isoflavones, especially genistein, reduce menopausal symptoms and menopause-related diseases like cardiovascular, osteoporosis, obesity, diabetes, anxiety, depression, and breast cancer [8,9,10]. They are called estrogen-like molecules or non-steroidal estrogens because of their similarity to 17-β-estradiol and their capability of binding to estrogen receptors [11]. Lower levels of estrogen in postmenopausal women lead to lower calcium availability and subsequent bone decalcification and osteoporosis [12], while short-term administration of isoflavones was found to positively affect bone metabolism [13]. Numerous studies have been conducted to investigate the impact of isoflavone intake on thyroid hormones in men, as well as its effect on prostate cancer [14,15]. The most recent research on daidzein has implications for its effect on cardiovascular risk reduction, and research on glycitein focuses on its bioavailability and its role in angiogenesis and invasion of malignant glioma cells [16]. Although there are contradictory studies on the adverse impact of long-term consumption of foods rich in isoflavones [17,18,19], the general impression is that their moderate use has a number of benefits for human health. For that reason, they are very common additions to dietary supplements. The first step for using isoflavones in the food and pharmaceutical industry is their extraction from plant material. Organic solvent extraction is the main method used to extract phenolics, and the most commonly used solvents are methanol and acetonitrile with different proportions of water. In addition to the solvent type, the extraction efficiency is affected by various factors, such as the matrix properties of the plant part, temperature, pressure, and time [20]. The conventional extraction procedure typically takes several hours to overnight and is usually performed in an ultrasonic bath or an orbital shaker. It has several disadvantages: Firstly, it is a time-consuming process that can lead to the oxidation and loss of unstable polyphenolic compounds. Additionally, most organic solvents used in the process are carcinogenic, necessitating their removal before obtaining the final extract. This requires additional purification steps that influence the total process cost. However, there is still a possibility that some residues of these solvents may remain in the final product. Legal limitations for solvent residues and restrictions on the use of conventional organic solvents are becoming more and more rigorous, especially in the fields of the food and pharmaceutical industry. Consequently, there is a growing imperative to explore novel methods for extracting polyphenolic compounds that are both environmentally acceptable and highly efficient [21]. Possible alternatives represent supercritical fluid extraction, ultrasound-assisted extraction, microwave-assisted extraction (MAE), subcritical water extraction, and enzyme-assisted subcritical water extraction, which, due to their simplicity, have shorter extraction times as well as reduced organic solvent consumption [22,23,24,25]. This study investigates the impact of microwave-assisted extraction on the isolation of isoflavones from soybean flour, as well as the antioxidant activity of the obtained extracts. By adjusting the temperature and power of the microwave reactor during different periods, the aim was to determine the most efficient extraction method. Microwave-assisted extraction is considered an environmentally friendly technique, requiring minimal or no solvent use at all. The reaction mixture is heated by direct interaction with free molecules present in the system, breaking down the cell wall and releasing bioactive components, improving the extraction process. Compared to traditional methods, microwave-assisted extraction tends to produce higher yields, reduce processing time, and minimize energy and solvent usage [25,26]. With its ability to destroy cell walls and facilitate the extraction of polyphenols, MAE represents a promising approach for the isolation of valuable compounds from plant sources [27]. Even though a commercially available microwave is a useful and convenient piece of equipment, the majority of research has been performed using household microwave units, which do not provide information about the final temperature achieved in the sample during treatment for the given power, do not provide any mixing during treatment, and usually only have preset microwave power levels available to the user. The objective of this research was to harness the benefits of a fully controlled and equipped microwave reactor system to enhance the extraction of isoflavones from soybean flour and to improve the antioxidant activity of the obtained extracts. Conventional maceration and ultrasound-assisted extraction were also applied in order to compare their effects to the effects of microwave energy regarding total phenols, total flavonoids, and antioxidant activity of the extracts.

## 2. Results

### 2.1. Total Phenol Content and Total Flavonoid Content

**Figure 2:**
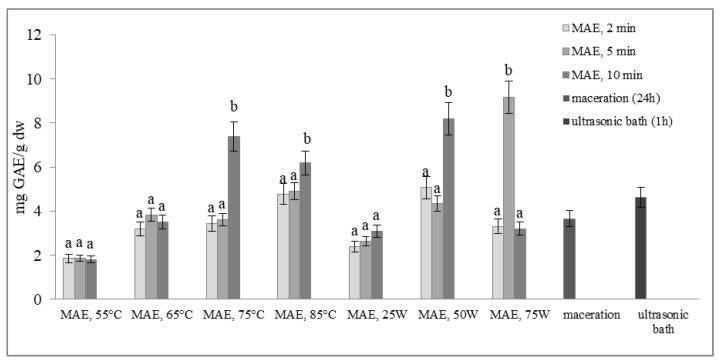
Total phenol content (mg GAE/g dw) of extracts obtained from defatted soy flour, influenced by different extraction procedures. Values with different superscripts for each applied procedure differ significantly (*p* < 0.05).

**Figure 3:**
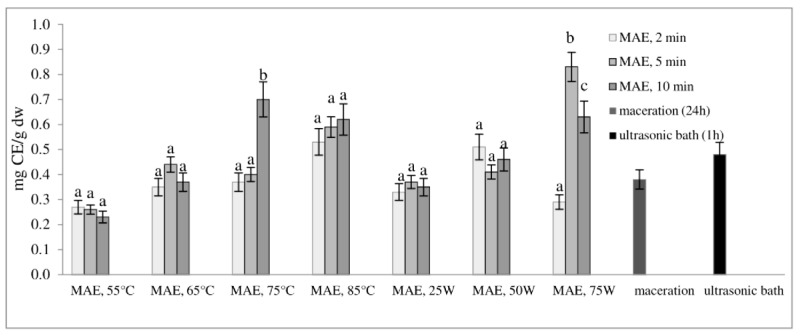
Total flavonoid content (mg CE/g dw) of extracts obtained from defatted soy flour influenced by different extraction procedures. Values with different superscripts for each applied procedure differ significantly (*p* < 0.05).

Figure 2 and Figure 3 show the total phenol content (TPC) and total flavonoid content (TFC) of extracts obtained from defatted soybean flour, depending on extraction conditions. The results of microwave-assisted extraction by varying the power of the microwave irradiation or the temperature at different time intervals are presented and compared to maceration and extraction in an ultrasonic bath. TPC and TFC increase in the same time interval, either with an increase in temperature or with an increase in the microwave power. The highest extraction efficiency, obtained by varying the temperature at different time intervals, was achieved at 75 °C, at which both TPC and TFC increased more than twice in just 10 min of extraction (from 3.61 to 7.37 mg GAE/g dw and from 0.23 to 0.70 mg CE/g dw, respectively).

With a further increase in temperature beyond 75 °C, TPC and TFC begin to decrease. Our experiment also shows that by modulating the power of microwave irradiation at different periods, there was a noticeable increase in total phenol and total flavonoid content as the power increased. This correlation is similar to the variation observed in temperature. The optimal condition for achieving the highest extraction efficiency was at the power of microwave irradiation of 75 W for only 5 min of extraction. With the extraction time extended further, at the same power of microwave irradiation, the temperature approached the pre-set maximum of 120 °C, causing a decrease in TPC and TFC. This can be explained by the degradation of polyphenols as thermolabile compounds.

Research findings indicate that regulating the power of microwave irradiation proved to be a more efficient method for the extraction of polyphenolic compounds from soy flour in comparison to temperature control of the reactor. This may be due to short interruptions of microwave irradiation at the moment of reaching the set temperature (55, 65, 75, or 85 °C). By controlling the irradiation power, the extraction process is more efficient due to a continuous effect of the microwave rays and uniform heating of the mixture (a comprehensive description of temperature variation and microwave irradiation variations for each case, along with a specific example for each operating mode of the microwave reactor, is provided in the Supplementary Materials). By further comparison of the most optimal microwave treatment conditions, namely 75 °C for 10 min and 75 W for 5 min, it can be deduced that both methods surpass conventional extraction techniques, such as maceration and extraction in an ultrasonic bath. The efficiency of both methods is superior to classic maceration because it shortens the extraction time by 144 or 288 times. The benefits of microwave-assisted extraction are further evidenced by the increased levels of total phenolic content and total flavonoid content observed under controlled power and temperature conditions. In particular, TPC and TFC levels were almost three times higher under controlled power (from 3.66 to 9.16 mg GAE/g dw and from 0.38 to 1.13 mg CE/g dw) and twice as high under controlled temperatures (from 3.66 to 7.37 mg GAE/g dw and from 0.38 to 0.70 mg CE/g dw) when compared to maceration. Microwave-assisted extraction also showed an advantage over extraction in an ultrasonic bath by not only shortening the extraction time by 6 to 12 times but also by doubling the amount of isolated TPC and TFC. These results demonstrate the clear advantages of microwave-assisted extraction for the extraction of polyphenolic compounds.

### 2.2. Antioxidant Activity of Extracts

**Figure 4:**
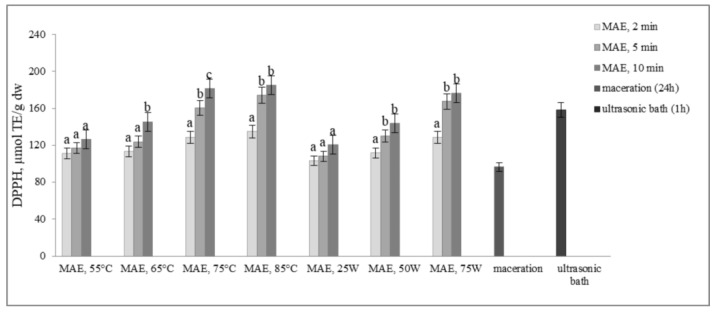
Antioxidant activity by DPPH assay (µmol TE/g dw) of extracts obtained from defatted soy flour influenced by different extraction procedures. Values with different superscripts for each applied procedure differ significantly (*p* < 0.05).

**Figure 5:**
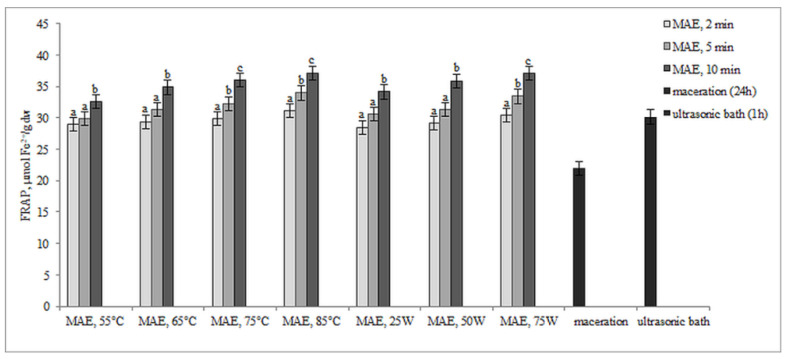
Antioxidant activity by FRAP assay (µmol Fe^2+^/g dw) of extracts obtained from defatted soy flour influenced by different extraction procedures. Values with different superscripts for each applied procedure differ significantly (*p* < 0.05).

The results presented in Figure 4 and Figure 5 illustrate the antioxidant activity of extracts obtained from defatted soy flour samples using various methods. Our findings reveal a strong linear correlation between TPC, TFC, and DPPH, as well as between TPC, TFC, and FRAP, indicating that higher levels of microwave power or temperature result in increased antioxidant activity.

However, an exception was found in our study when samples were subjected to controlled microwave irradiation of 75 W for 10 min, resulting in an increase in antioxidant activity, determined by both methods, despite decreases in TPC and TFC. Extracts obtained by extraction in the ultrasonic bath also showed remarkably high antioxidant activity, only about 12.6% less than MAE extracts, although the levels of TPC and TFC were very low. Based on these findings, it can be inferred that some non-phenolic antioxidant compounds were extracted. This is likely because ultrasound has the ability to disrupt plant cell walls, which in turn enhances solvent penetration.

### 2.3. Individual Isoflavone Content

Table 1, Table 2 and Table 3 present the content of six individual isoflavones, three aglycones, and three glucosides monitored in extracts obtained from defatted soy flour based on the extraction conditions. By examining Table 1, it is evident that manipulating the temperature of microwave irradiation impacts the extraction efficiency of isoflavones. In particular, an increase in temperature from 55 °C to 65 °C within a 2 min interval results in a statistically significant increase in extraction efficiency for four out of six monitored isoflavones. While further temperature increases to 75 °C do not typically yield significant changes, they become apparent again when the temperature is raised to 85 °C. Additionally, lengthening the extraction time to 5 min highlights a noticeable difference in extraction efficiency when the temperature is increased from 55 °C to 65 °C. Between the temperatures of 65 °C and 75 °C, there is no statistically significant difference in the extraction efficiency, but it occurs as the temperature increases to 85 °C.

The results also indicate that increasing the extraction time to 10 min and raising the temperature from 75 °C to 85 °C improved the extraction efficiency of five of the six monitored isoflavones. However, despite this improvement, there was no statistically significant difference in the total amount of isoflavones extracted. We did observe a statistically significant decrease in the extraction efficiency of glycitein with an increased extraction time and temperature. This decrease in efficiency corresponds with the obtained TPC and TFC, which could be attributed to the degradation of isoflavones. By observing the total quantity of six extracted isoflavones, as time progressed at the same extraction temperature, there were no significant differences in extraction efficiency, except between five and ten minutes at 75 °C. Nevertheless, a combination of elevated time and temperature resulted in a synergistic impact that amplified the extraction efficiency.

Table 2 illustrates that altering the power of microwave irradiation within the same extraction time results in statistically significant differences. The extraction efficiency is observed to increase when the power is raised from 25 W to 50 W, similar to the impact of temperature variation. However, a further increase in power from 50 W to 75 W leads to a decrease in extraction efficiency. The likely cause for this is the rise in temperature of the reaction mixture, which is accelerated by the increase in power and ultimately results in the degradation of isoflavones.

Upon analyzing the individual isoflavone content (Table 1, Table 2 and Table 3), it was found that certain isoflavones are predominantly found in the glucoside form (daidzin and genistin) rather than their accompanying aglycones, daidzein and genistein. An exception is the extremely high glycitein content compared to its corresponding glucoside form, glycitin. This result could be very significant for several reasons. First, this variety can be characterized as a variety with a high content of glycitein, whose MAE extraction conditions we optimized. Also, the latest research on glycitein focuses on its role in angiogenesis and invasion of malignant glioma cells [16]. Therefore, the results of this research can represent a good basis for further analysis of the extracts from the aspects of their purity, stability, and further purification.

## 3. Discussion

Our research on the microwave-assisted extraction of total phenolics and total flavonoids aligns with previous studies [28,29], suggesting that increasing the temperature or microwave power enhances the extraction efficiency of bioactive compounds from plant materials. In general, the efficiency of MAE, in addition to increasing the extraction yield, is also reflected in a significant shortening of the extraction time (in our study, 288 times shorter time compared to maceration and 12 times shorter compared to sonication). These results are consistent with numerous studies. The findings of Gallo et al. showed that the efficiency of the microwave-assisted extraction was, on average, four times higher than the efficiency of the sonication extraction [26]. In the work of Álvarez et al., microwave pretreatment increased the yield of polyphenols by 57% [30]. In the research of Silveira da Rosa et al., MAE at a higher temperature (86 °C) with a short extraction time (3 min) increased the yield of TPC from olive leaves by 82% when compared to maceration [31]. In another research, Zhang et al. compared MAE with the traditional maceration and Soxhlet extraction methods for the extraction of phenolic compounds from the waste of Sterculia nobilis fruit. Extraction efficiency and the antioxidant activity by MAE were 2.24 times and 3.93 times higher than those acquired by the maceration method and Soxhlet extraction, respectively. In terms of extraction time, MAE (37.37 min) required significantly less time than the maceration method (24 h) and Soxhlet extraction (4 h) [32]. The yields of the extracted compounds obtained by microwave irradiation were several times higher compared with those obtained by the traditional Soxhlet or shake-flask extraction methods [25]. However, in our study, when the temperature or microwave power exceeds 75 °C or 75 W and the extraction time is extended from 5 to 10 min, the extraction efficiency decreases. These results are consistent with Belwal et al.’s findings, indicating that high temperatures may cause thermal degradation of polyphenols [33]. Another study by Shahid et al. suggested that oxidation of polyphenols at elevated temperatures could also lead to a reduction in total phenolic content and antioxidant activity [34]. Similarly, Antoni and Farid, in their review, reported that while high power may enhance the heating effect and reduce microwave irradiation time, it may also lead to the degradation of thermolabile components [35]. In contrast, increasing the microwave power and extending the extraction time had a positive effect on the antioxidant activity (DPPH and FRAP) of the obtained extracts. This increase may be attributed to the extraction of other non-phenolic compounds with antioxidant properties, such as vitamins or sugars, under these specific conditions [36]. Soy, in addition to isoflavones and polyphenolic acids, also contains significant amounts of carotenoids and tocopherols, which also reveal antioxidant activity [37,38,39]. Recent studies also have found that with further increases in microwave power or temperature, DPPH and FRAP tend to increase, while TPC and TFC decrease [40,41]. Our experimental results showed that controlling the power of microwave irradiation was more effective because the temperature limit was not exceeded during the given extraction times, ensuring continuous microwave irradiation. In contrast, when using the temperature-controlled mode, the microwave action was intermittently stopped once the set temperature was reached, particularly during longer extraction times, resulting in inefficient, discontinuous microwave irradiation. This lack of continuous irradiation led to less efficient extraction. At higher temperatures, MW irradiation was applied more frequently by the reactor and for longer durations than at lower temperatures, which resulted in higher extraction efficiency (the amount of irradiation is bigger). Here, when the set temperature was reached, irradiation either caused or was significantly reduced, particularly at lower temperatures, where minimal irradiation or small irradiation was required to maintain the temperature (Supplementary Materials). As a result, operating with constant irradiation power generally provided better extraction results.

The increased antioxidant activity of samples extracted using microwaves, compared to conventional maceration, supports the fact that microwave irradiation reaches the interior of the cell, causing the heating of the intracellular material and leading to the partial destruction of the cell wall followed by the release of bioactive components [27]. Our research indicates that in defatted soy flour, isoflavones are predominantly present in the form of glucosides (daidzin and genistin) rather than their accompanying aglycones, daidzein and genistein. Their subsequent conversion from conjugated forms to bioactive aglycones occurs as a result of heating, enzyme activity, etc. This is also consistent with other reports that conjugated forms, particularly malonylglucosides of isoflavones, are predominantly found in soy-based foods [42,43,44]. Furthermore, increasing temperature or microwave power while extending extraction time was observed to positively affect the extraction of four of the six monitored isoflavones. These results align with Terrigar et al.’s research, which demonstrated a threefold increase in the concentration of extracted isoflavones for genistin and daidzin when the temperature rose from 55 °C to 73 °C and the extraction time increased from 4 min to 8 min. Additionally, extending the extraction time above 8 min resulted in no significant differences in the isoflavone concentration [45]. It has been noted that prolonged extraction at high temperatures can result in the degradation of isoflavones [28,29]. A comparison between ultrasonic bath extraction and maceration (Table 3) revealed that ultrasonic extraction showed a clear advantage over maceration in extracting five of the six monitored isoflavones. This finding is in line with the study of Courage et al. indicating that, in addition to microwave irradiation, ultrasound waves are effective in breaking cell walls and aiding in the extraction of internal components [46]. In their review, Sojata et al. compared many studies examining soy isoflavones. Most of them have been focused on monitoring TPC and TFC in soybeans and soy flour, as well as the levels of daidzein and genistin and their aglycones [47]. However, there are little data on glycine and glycitein. Our research revealed extremely high glycitein content compared to the corresponding form of glucoside, glycitin. This finding could be very significant, primarily due to recent research that highlighted the role of glycitein in angiogenesis and invasion of malignant glioma cells [16], and its extraction from plant material is the first step in its application for pharmaceutical purposes.

## 4. Materials and Methods

### 4.1. Plant Material

The soybean variety Laura (yellow grain) was used in the experiment. This variety was selected for several reasons. It is characterized by the lack of the Kunitz trypsin inhibitor protein (an inhibitor with significant antinutritive effects), which makes it very useful. Also, the results of our previous research on this variety showed a positive influence of microwave pretreatment on the extraction of polyphenolic acids [48]. A representative sample of 2 kg was provided from the Maize Research Institute, Zemun polje, and represents a variety that is part of their regular annual cultivation. Grains were milled and sieved through a laboratory sieve of 500 μm diameter. The obtained flour was defatted in petrol ether in a Soxhlet extractor for 4 h. The samples were stored in the freezer before extraction.

### 4.2. Chemicals and Reagents

Analytical standards genistein (≥99%), glycitein (≥99%), daidzein (≥99%), genistin (≥99%), glycitin (≥99%), daidzin (≥99%), gallic acid (≥97.5%), and (+)-catechin (≥98%) were purchased from Sigma-Aldrich (St. Louis, MO, USA). HPLC methanol (≥99.9%), analytical grade water, 2,2-diphenyl-1-picrylhydrazyl (DPPH), 6-hydroxy-2,5,7,8-tetramethylchroman-2-carboxylic acid (TROLOX, 97%), and acetonitrile (≥99.9%) were purchased from Sigma-Aldrich (St. Louis, MO, USA); 2,4,6-Tris (2-pyridyl)-s-triazine (TPTZ, ≥99%) was purchased from Fluka (Büchi, St. Gallen, Switzerland); the Folin–Ciocalteu reagent was purchased from Reagecon (Clare, Ireland). All other solvents and chemicals were p.a. or higher purity and used as received without further purification.

### 4.3. Methods

#### 4.3.1. Extraction of Plant Material

The extraction of bioactive compounds from previously grounded and defatted soy flour was performed according to the following procedure: 0.5 g of flour was weighed on an analytical balance and mixed with a solvent consisting of methanol and 0.1% hydrochloric acid in a ratio of 85:15% at a volume of 10 mL. The mixture was further subjected to microwave extraction by varying different parameters. The extraction procedure was performed in a microwave reactor system (Microwave Synthesis Reactor, Monowave 300, Anton Paar, Graz, Austria). This reactor has the capability to operate in two modes: temperature-controlled mode and power-controlled mode. In the temperature-controlled mode, the desired temperature and extraction time are set, and the device automatically adjusts the power to reach the set temperature. In this mode, the device chooses the irradiation power itself, starting with a high power level and then gradually decreasing it as the temperature approaches the set point. In the power-controlled mode, power and temperature limits are set since the reaction mixture is heated during the action of microwave irradiation (the extraction time was also set). The temperature limit prevents overheating of the reaction mixture. In this mode, the device radiates with constant power until it reaches the set temperature and maintains it with that power. In both regimes, microwave power and temperature are closely related, and the speed of temperature growth under the action of microwave irradiation depends on several factors, primarily on the matrix itself and the solvent used. The first part of the experiment involved varying the temperature, with extractions performed at 55 °C, 65 °C, 75 °C, and 85 °C for 2, 5, and 10 min. The temperature was kept constant, and the microwave power was increased until the assigned temperature was reached. The second part of the experiment involved changing the microwave power at 25 W, 50 W, and 75 W in given time intervals of 2, 5, and 10 min. To prevent the degradation of bioactive compounds in soybean flour, the maximum temperature was set at 120 °C. An extraction procedure by maceration was performed in parallel, with the mixture extracted at room temperature in a shaker at 300 rpm for 24 h, as well as in an ultrasonic bath (A-Sonic, 400 Hz), lasting 60 min. Further, all extracts were centrifuged at 15.000× *g* for 15 min, and supernatants were filtered through 0.45 µm PTFE filters and stored in a refrigerator until analysis.

#### 4.3.2. Total Phenol Content (TPC)

The determination of total phenol content (TPC) in prepared extracts was performed spectrophotometrically using the Folin–Ciocalteu reagent. A total of 50 µL of previously obtained plant extracts were mixed with 3 mL of water, 0.25 mL of Folin–Ciocalteu reagent (diluted with water in a ratio of 1:1), and 750 µL of 20% Na_2_CO_3_. After incubating for 8 min at room temperature, another 950 µL of water was added to test tubes, and mixtures were incubated for 120 min at room temperature, and the absorbance was read at 765 nm (UV-VIS 2100 Spectrophotometer, Shimadzu, Kyoto, Japan). TPC was determined by correlating the absorbance values of the samples with the calibration curve constructed using gallic acid. The stock solution of gallic acid was prepared in methanol at a concentration of 1.0 mg/mL. The working solutions were prepared by diluting the stock solution with methanol to concentrations of 25, 50, 75, 100, 150, 200, and 250 µg/mL. The results were expressed as mg gallic acid equivalents (GAEs) per gram of dry weight (mg GAE/g dw).

#### 4.3.3. Total Flavonoid Content (TFC)

The determination of total flavonoid content (TFC) in prepared extracts was performed spectrophotometrically, according to the Woumbo et al. method [49] with slight modifications. Briefly, 0.5 mL of the previously obtained plant extracts were mixed with 2.5 mL of distilled water and 150 µL of 5% NaNO_2_ solution. After incubating for 6 min at room temperature, 300 µL of 10% AlCl_3_×6H_2_O was added to test tubes and allowed to incubate for another 5 min before adding 1 mL of 1 M NaOH solution. The mixture was brought to 5 mL with distilled water, mixed, and the absorbance was measured immediately at 510 nm (UV-VIS 2100 Spectrophotometer, Shimadzu, Japan) against the blank (the same mixture without the sample). At the same time, the calibration curve of (+)-catechin was constructed in the concentration range of 10 to 1000 µg/mL. The results were expressed in milligrams of (+)-catechin equivalents per gram of dry weight (mg CE/g dw).

#### 4.3.4. Antioxidant Activity

Antioxidant activity depends on many factors, such as environmental conditions, physiological processes in the plant itself, as well as processing conditions. To evaluate the antioxidant activity of various substances of plant origin as efficiently as possible, it is necessary to combine several different methods. In this research, we assessed antioxidant activity using two different assays, 2,2-di(4-tert-octylphenyl)-1-picrylhydrazyl (DPPH) and ferric reducing antioxidant power (FRAP). While the FRAP assay is based on the transfer of one electron, DPPH belongs to the group of mixed tests, including the transfer of both a hydrogen atom and an electron [50]. Both are spectrophotometric methods based on the color change reaction of reagents in contact with compounds that have antioxidant capacity.

The determination of antioxidant activity of extracts by the DPPH method was carried out according to the Payum et al. method [51] with slight modifications. Previously obtained soybean flour extracts (used for TPC and TFC measurements) were diluted 10 times with 85% methanol. The solution of DPPH in methanol (0.1 mM) was prepared and kept in a dark bottle, covered in aluminum foil, for approximately 30 min. Then, 200 μL of the diluted extracts were mixed with 3.8 mL of 0.1 mM DPPH reagent. Color development from purple to yellow was achieved by incubating the mixture in the dark at room temperature for 30 min. At the same time, the standard curve of the TROLOX reagent was prepared in the concentration range of 0 to 1000 µmol/L. The antioxidant activity was determined by reading the absorbance at 517 nm against methanol in relation to the calibration curve of TROLOX and was expressed as μmol TE/g dw.

The determination of antioxidant activity of extracts by the FRAP method was carried out following the Benzie and Strain method [52]. The same 10-time diluted soyflour extracts were used. A total of 4.5 mL of FRAP reagent and 150 μL of the diluted extract were incubated for 30 min at 37 °C. The absorbance of blue-colored solutions was measured at 593 nm against a blank sample. The FRAP reagent consists of 2.5 mL of 2,4,6-tripyridyl-s-triazine (TPTZ) reagent, 2.5 mL of 20 mM FeCl_3_×6H_2_O solution, and 25 mL of 0.3 M acetate buffer (pH 3.6). A calibration curve with FeSO_4_×7H_2_O was constructed concurrently in the concentration range from 0 to 1000 µmol/mL. The values were expressed as μmol Fe^2+^/g dw.

#### 4.3.5. HPLC Analysis of Isoflavones

The quantification of isoflavones in soybean flour was carried out by liquid chromatography on the HPLC-DAD Nexera XR system (Shimadzu, Japan) with an autosampler and quaternary pump. For the separation of isoflavones, a Zorbax SB C18 column (250 × 4.6 mm, id 5 μm) (Agilent Corporation, Santa Clara, CA, USA) was used. The column was thermostatically controlled at 25 °C, the injection volume was 20 µL, and the flow rate was set to 1 mL/min. The mobile phase consisted of two solvents: 0.1% formic acid (solvent A) and methanol (solvent B). The solvent gradient was as follows: 0 min 5% B, 25 min 30% B, 35 min 40% B, 40 min 48% B, 50 min 70% B, 55 min 100% B, 65 min 5% B, re-equilibration time 10 min. The wavelength used for the quantification was 254 nm. The identification of isoflavones was accomplished by comparing the retention time of the peaks to those of standard compounds (Figure S1). Stock solutions of daidzin, daidzein, genistin, genistein, glycitin, and glycitein were prepared in a methanol–water mixture (75:25, *v*/*v*) at a concentration of 1.0 mg/mL. The working solutions were prepared by diluting the stock solutions with a methanol–water mixture to concentrations of 10, 25, 50, 100, and 150 µg/mL. The quantitation of isoflavones was based on calibration curves built for each compound (Figure S2). The linear correlation coefficients were 0.995 or higher. The results were expressed as µg per g of dry weight (µg/g dw).

### 4.4. Statistical Analysis

All the experiments were performed in triplicate, and the results were expressed as the mean ± standard deviation (SD). TPC, TFC, DPPH, and FRAP data, as well as data from extraction by maceration and in an ultrasonic bath, were subjected to one-way analysis of variance (ANOVA) to compare the effects of temperature and microwave power. Individual isoflavones data were subjected to a two-way analysis of variance (ANOVA) to evaluate the effect of temperature, microwave power, time, and their interaction. Differences between means were determined using Tukey’s HSD (honestly significant difference) test at the significance level of *p* < 0.05. All statistical analyses, including calculations, were conducted using the Statistica 13.3 software package (Tibco Inc., Palo Alto, CA, USA).

## 5. Conclusions

The main objective of this research was to examine the effectiveness of microwave-assisted extraction of isoflavones from soy flour. By manipulating variables such as microwave strength, temperature, and duration, we were able to determine the optimal parameters for extracting individual isoflavones with maximum efficiency (irradiation power of 75 W for 5 min). Our research revealed an extremely high glycitein content in extracts, compared to its glucoside form, glycitin. This finding could be highly significant, considering the growing recognition of glycitein’s potential role in medical applications, including its impact on angiogenesis and the invasion of malignant cells. The extraction of glycitein from plant materials represents a crucial step towards its potential use in pharmaceuticals. In addition, our research revealed a positive correlation between microwave exposure and the levels of total phenols, total flavonoids, and antioxidant activity in the obtained extracts. These results show that MAE is a reliable and efficient method for the extraction of isoflavones from soybeans, offering a significantly reduced extraction time while obtaining high yields and using a very small amount of organic solvents.

## Figures and Tables

**Table 1:** Influence of temperature (Te), time (Ti), and their interaction (TexTi) of microwave irradiation on the content of six isoflavones in extracts obtained from defatted soybean flour (µg/g dw).

*n* = 3 (µg/g dw)	Time	Temperature				Level of Significance		
Isoflavones		55 °C	65 °C	75 °C	85 °C	Te	Ti	TexTi
Daidzin	2 min	30.68 ± 0.56 ^a^	80.65 ± 0.79 ^bA^	84.28 ± 1.20 ^bA^	116.62 ± 2.63 ^cA^	***	***	***
	5 min	28.29 ± 0.24 ^a^	89.82 ± 0.07 ^bB^	93.02 ± 0.66 ^bB^	121.52 ± 2.12 ^cA^			
	10 min	30.57 ± 0.94 ^a^	105.51 ± 1.66 ^bC^	107.09 ± 2.91 ^bC^	156.28 ± 3.03 ^cB^			
Glycitin	2 min	0.00 ± 0.00 ^aA^	0.81 ± 0.05 ^bA^	2.60 ± 0.47 ^cB^	3.30 ± 0.14 ^d^	***	***	***
	5 min	0.00 ± 0.00 ^aA^	1.55 ± 0.08 ^bB^	1.76 ± 0.28 ^bA^	3.37 ± 0.37 ^c^			
	10 min	0.71 ± 0.22 ^aB^	3.04 ± 1.05 ^bC^	2.87 ± 0.05 ^bB^	3.71 ± 0.07 ^c^			
Genistin	2 min	30.71 ± 1.52 ^a^	91.59 ± 1.00 ^bA^	88.26 ± 2.64 ^bA^	122.38 ± 4.04 ^cA^	***	***	***
	5 min	29.90 ± 0.91 ^a^	99.73 ± 1.69 ^bA^	103.34 ± 1.73 ^bB^	126.38 ± 3.08 ^cA^			
	10 min	32.90 ± 0.70 ^a^	117.95 ± 2.88 ^bB^	124.01 ± 0.41 ^bC^	157.51 ± 7.10 ^cB^			
Daidzein	2 min	5.43 ± 0.05 ^cC^	5.25 ± 0.46 ^bcB^	4.50 ± 0.28 ^bB^	1.90 ± 0.09 ^aA^	***	***	***
	5 min	4.35 ± 0.10 ^bB^	4.97 ± 0.13 ^bB^	2.67 ± 0.25 ^aA^	2.40 ± 0.57 ^aA^			
	10 min	1.34 ± 0.17 ^aA^	2.84 ± 0.18 ^bA^	5.08 ± 0.36 ^cB^	5.34 ± 0.11 ^cB^			
Glycitein	2 min	141.20 ± 6.34 ^a^	449.90 ±3.01 ^bcAB^	438.19 ± 4.09 ^bA^	495.90 ± 15.77 ^c^	***	NS	***
	5 min	146.03 ± 4.25 ^a^	477.97 ± 5.46 ^bB^	443.73 ± 8.94 ^bA^	487.59 ± 55.32 ^bc^			
	10 min	110.98 ± 1.79 ^a^	418.67 ± 21.25 ^bA^	559.99 ± 5.44 ^cB^	452.15 ± 8.17 ^b^			
Genistein	2 min	2.77 ± 0.05 ^a^	3.28 ± 0.05 ^aA^	3.13 ± 0.09 ^aA^	6.02 ± 0.35 ^bA^	***	***	***
	5 min	2.62 ± 0.11 ^a^	3.37 ± 0.07 ^abA^	4.06 ± 0.24 ^bB^	6.48 ± 0.48 ^cA^			
	10 min	3.43 ± 0.26 ^a^	5.71 ± 0.26 ^bB^	4.11 ± 0.17 ^aB^	8.41 ± 0.66 ^cB^			
Total	2 min	210.78 ± 8.35 ^a^	631.48 ± 4.47 ^b^	620.96 ± 7.02 ^bA^	746.11 ± 22.06 ^c^	***	***	***
	5 min	211.19 ± 5.22 ^a^	677.41 ± 6.22 ^b^	648.58 ±10.65 ^bA^	747.73 ± 60.03 ^c^			
	10 min	179.94 ± 1.27 ^a^	653.72 ± 25.95 ^b^	803.15 ± 8.24 ^cB^	783.40 ± 17.58 ^c^			

^a, b, c, d^ Means within the same row with different superscripts differ significantly (*p* < 0.05); ^A, B, C^ means within the same column with different superscripts differ significantly (*p* < 0.05); *** *p* < 0.001; NS—not significant.

**Table 2:** Influence of power (P), time (Ti), and their interaction (PxTi) of microwave irradiation on the content of six isoflavones in extracts obtained from defatted soybean flour µg/g dw.

*n* = 3 (µg/g dw)	Time	Power			Level of Significance		
Isoflavones		25 W	50 W	75 W	P	Ti	PxTi
Daidzin	2 min	59.35 ± 2.97 ^aA^	117.05 ± 4.11 ^cA^	90.54 ± 0.29 ^bA^	***	***	***
	5 min	71.17 ± 2.42 ^aB^	122.50 ± 3.27 ^bA^	184.24 ± 3.84 ^cC^			
	10 min	81.42 ± 7.26 ^aB^	147.24 ± 4.45 ^cB^	131.94 ± 1.86 ^bB^			
Glycitin	2 min	0.00 ± 0.00 ^aA^	2.92 ± 0.10 ^cC^	1.94 ± 0.15 ^bB^	***	***	***
	5 min	0.70 ± 0.21 ^aB^	2.32 ± 0.17 ^bB^	7.31 ± 0.23 ^cC^			
	10 min	1.22 ± 0.15 ^C^	1.11 ± 0.08 ^A^	0.85 ± 0.02 ^A^			
Genistin	2 min	61.77 ± 4.29 ^aA^	127.30 ± 2.18 ^cA^	103.84 ± 1.12 ^bA^	***	***	***
	5 min	81.91 ± 1.03 ^aB^	130.51 ± 4.09 ^bA^	211.10 ± 3.37 ^cC^			
	10 min	95.01 ± 4.53 ^aC^	145.09 ± 4.74 ^cB^	132.26 ± 2.69 ^bB^			
Daidzein	2 min	0.00 ± 0.00 ^a^	0.00 ± 0.00 ^aA^	1.35 ± 0.07 ^bA^	***	***	***
	5 min	0.00 ± 0.00 ^a^	0.50 ± 0.08 ^bB^	1.88 ± 0.11 ^cB^			
	10 min	0.00 ± 0.00 ^a^	0.96 ± 0.05 ^bC^	1.32 ± 0.10 ^cA^			
Glycitein	2 min	196.73 ± 10.94 ^aA^	514.26 ± 13.47 ^cB^	434.37 ± 1.92 ^bB^	***	***	***
	5 min	323.01 ± 6.56 ^aB^	463.80 ± 15.12 ^bA^	551.84 ± 9.78 ^cC^			
	10 min	349.40 ± 15.60 ^aB^	534.12 ± 13.80 ^bB^	366.74 ± 10.35 ^aA^			
Genistein	2 min	3.69 ± 0.10	4.18 ± 0.16 ^A^	3.43 ± 0.14 ^A^	***	***	***
	5 min	3.42 ± 0.08 ^a^	5.16 ± 0.83 ^bA^	7.13 ± 0.32 ^cB^			
Total	10 min	3.87 ± 0.26 ^a^	10.46 ± 0.58 ^cB^	8.03 ± 0.20 ^bB^			
	2 min	321.55 ± 18.00 ^aA^	765.72 ± 19.81 ^cA^	635.47 ± 1.64 ^bA^	***	***	***
	5 min	480.21 ± 8.91 ^aB^	724.30 ± 21.55 ^bA^	963.52± 17.35 ^cB^			
	10 min	530.92 ± 27.55 ^aB^	838.02 ± 23.19 ^cB^	641.16± 15.09 ^bA^			

^a, b, c^ Means within the same row with different superscripts differ significantly (*p* < 0.05); ^A, B, C^ means within the same column with different superscripts differ significantly (*p* < 0.05); *** *p* < 0.001.

**Table 3:** The influence of different extraction procedures on the content of six isoflavones in extracts obtained from defatted soybean flour (µg/g dw).

Isoflavones	Maceration	Ultrasound Bath
daidzin	87.442 ± 0.74 ^a^	135.58 ± 1.62 ^b^
glycitin	0.62 ± 0.05 ^a^	3.21 ± 0.13 ^b^
genistin	99.13 ± 2.03 ^a^	140.95 ± 1.89 ^b^
daidzein	3.2 ± 0.08 ^b^	1.53 ± 0.13 ^a^
glycitein	468.43 ± 14.59 ^a^	454.31 ± 2.45 ^a^
genistein	2.99 ± 0.08 ^a^	6.71 ± 0.20 ^b^
Total	661.81	742.29

^a, b^ Means within the same row with different superscripts differ significantly (*p* < 0.05).

## Data Availability

Data are contained within the article and Supplementary Materials.

## Supplementary Materials

The following supporting information can be downloaded at: https://www.mdpi.com/article/10.3390/molecules29194685/s1, Figure S1: Chromatogram of six isoflavone standards in the concentration range from 25 to 150 µg/mL; Figure S2: Calibration curves of six isoflavone standards; Figure S3: Example of the temperature-controlled mode at 55 °C for 10 min, with a graphical representation of the irradiation power variations during the experiment; Figure S4: Example of the power-controlled mode at 50 W for 2 min, with a graphical representation of the temperature variations during the experiment; Table S1: Variation in microwave irradiation power in mode 1 (temperature-controlled mode) and temperature in mode 2 (power-controlled mode) at different extraction times.
