# Supplementary material for: Microwave Irradiation as a Powerful Tool for Isolating Isoflavones from Soybean Flour
Source: Molecules. 2024 Oct 2;29(19):4685. doi: 10.3390/molecules29194685 (PMC11477798; doi:10.3390/molecules29194685)
Supplement: Supplementary file 1 [file molecules-29-04685-s001.zip › Figure S1.pdf]

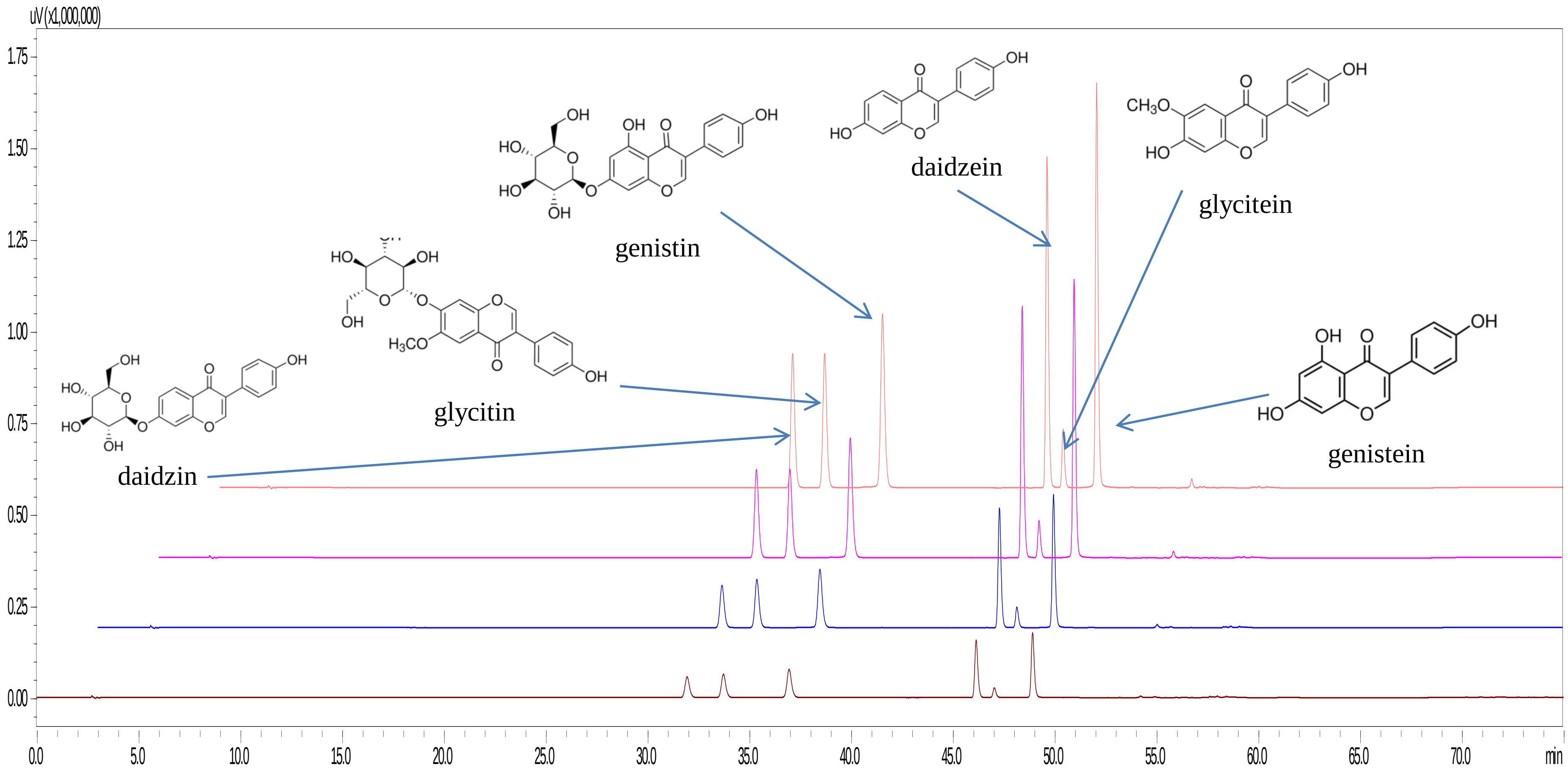

Figure S1: Chromatogram of six isoflavone standards in the concentration rang from 25 to 150 µg/ml
