# Supplementary material for: Microwave Irradiation as a Powerful Tool for Isolating Isoflavones from Soybean Flour
Source: Molecules. 2024 Oct 2;29(19):4685. doi: 10.3390/molecules29194685 (PMC11477798; doi:10.3390/molecules29194685)
Supplement: Supplementary file 1 [file molecules-29-04685-s001.zip › Figure S2.pdf]

Figure S2: Calibration curves of six isoflavone standards.

==== Shimadzu LabSolutions Calibration Curve ====

ID# : 1
Name : daidzin
Quantitative Method : External Standard
Function : f(x)=37978.6*x-63699.8
Rr1=0.9998248 Rr2=0.9996497 RSS=6.713127e+009
MeanRF: 3.650759e+004 RFSD: 1.213821e+003 RFRSD: 3.324847
FitType : Linear
ZeroThrough : Not Through
Weighted Regression : None
Detector Name : PDA

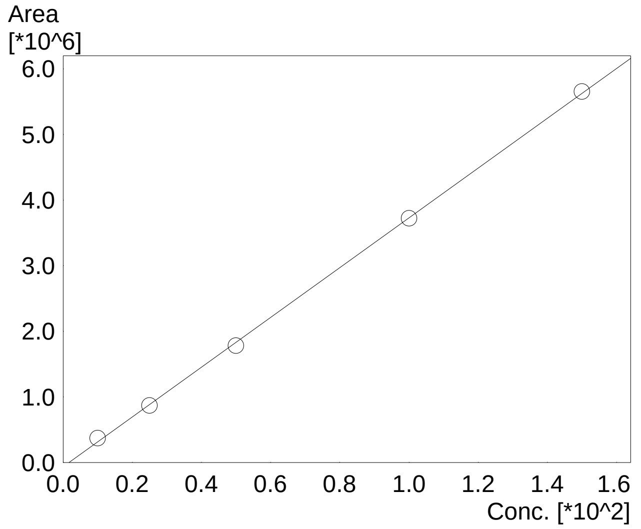

#	Conc.(Ratio)	MeanArea	Area
1	10	371159	371159
2	25	870589	870589
3	50	1783019	1783019
4	100	3722312	3722312
5	150	5657245	5657245

ID# : 2
Name : glicitim
Quantitative Method : External Standard
Function : f(x)=36652.1*x+75556.9
Rr1=0.9995995 Rr2=0.9991992 RSS=1.429737e+010
MeanRF: 3.883162e+004 RFSD: 1.816956e+003 RFRSD: 4.679062
FitType : Linear
ZeroThrough : Not Through
Weighted Regression : None
Detector Name : PDA

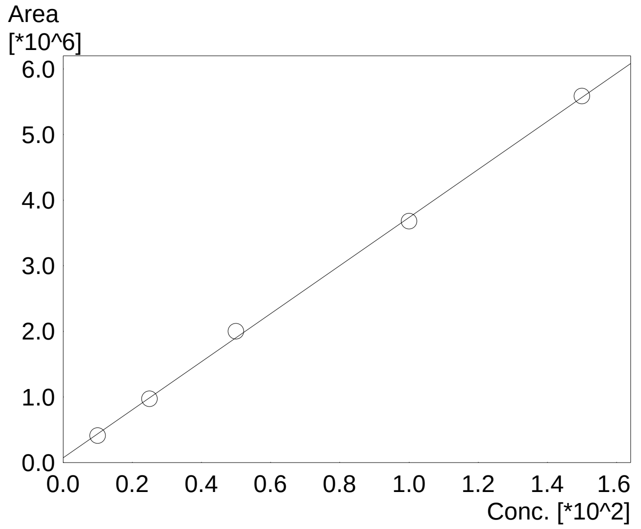

#	Conc.(Ratio)	MeanArea	Area
1	10	410676	410676
2	25	973620	973620
3	50	2004597	2004597
4	100	3681427	3681427
5	150	5585926	5585926

ID# : 3
Name : genistin
Quantitative Method : External Standard
Function : f(x)=51585.9*x-47552.6
Rr1=0.9997155 Rr2=0.9994311 RSS=2.011691e+010
MeanRF: 4.944107e+004 RFSD: 2.876533e+003 RFRSD: 5.818104
FitType : Linear
ZeroThrough : Not Through
Weighted Regression : None
Detector Name : PDA

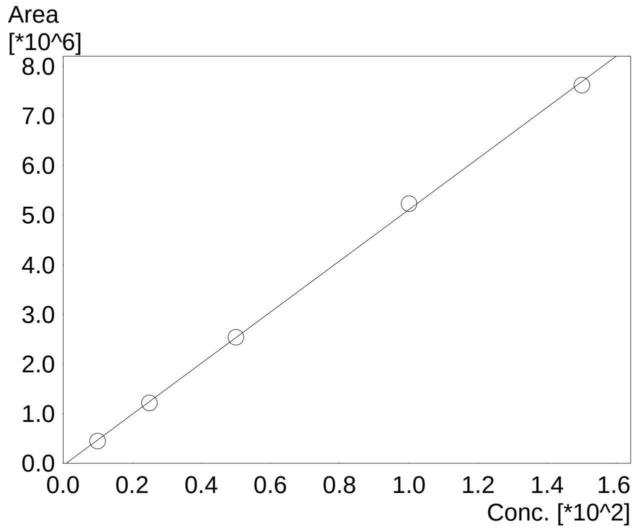

#	Conc.(Ratio)	MeanArea	Area
1	10	448870	448870
2	25	1213018	1213018
3	50	2537184	2537184
4	100	5227279	5227279
5	150	7617175	7617175

ID# : 4
Name : daidzein
Quantitative Method : External Standard
Function : f(x)=66179.4*x+185226
Rr1=0.9957971 Rr2=0.9916119 RSS=4.920006e+011
MeanRF: 7.051353e+004 RFSD: 4.382600e+003 RFRSD: 6.215262
FitType : Linear
ZeroThrough : Not Through
Weighted Regression : None
Detector Name : PDA

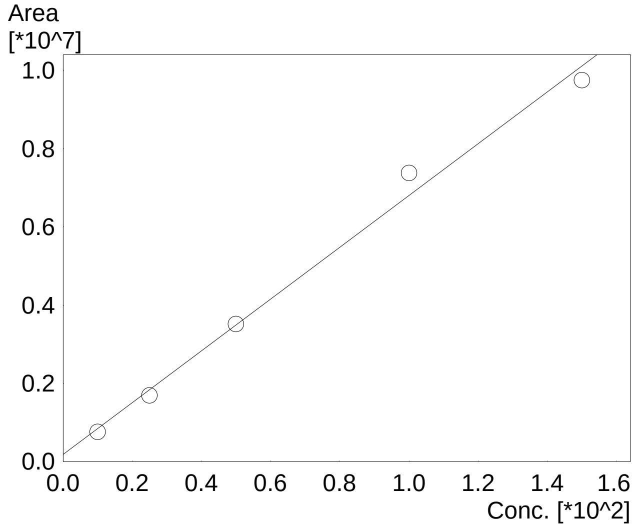

#	Conc.(Ratio)	MeanArea	Area
1	10	757739	757739
2	25	1691691	1691691
3	50	3516303	3516303
4	100	7378996	7378996
5	150	9751510	9751510

ID# : 5
Name : glicitein
Quantitative Method : External Standard
Function : f(x)=10927.6*x+12400.0
Rr1=0.9987439 Rr2=0.9974893 RSS=3.991409e+009
MeanRF: 1.143621e+004 RFSD: 7.685150e+002 RFRSD: 6.720016
FitType : Linear
ZeroThrough : Not Through
Weighted Regression : None
Detector Name : PDA

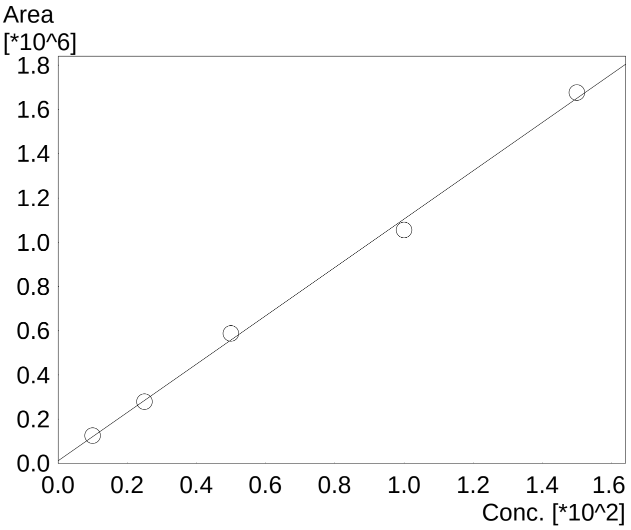

#	Conc.(Ratio)	MeanArea	Area
1	10	125847	125847
2	25	278070	278070
3	50	587296	587296
4	100	1055239	1055239
5	150	1676283	1676283

ID# : 6
Name : genistein
Quantitative Method : External Standard
Function : f(x)=79379.2*x-106045
Rr1=0.9997787 Rr2=0.9995575 RSS=3.704248e+010
MeanRF: 7.550197e+004 RFSD: 4.139814e+003 RFRSD: 5.483054
FitType : Linear
ZeroThrough : Not Through
Weighted Regression : None
Detector Name : PDA

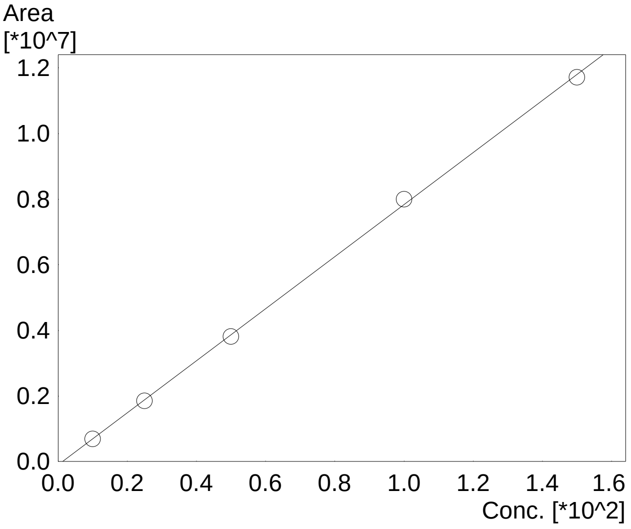

#	Conc.(Ratio)	MeanArea	Area
1	10	691953	691953
2	25	1852788	1852788
3	50	3808341	3808341
4	100	7993377	7993377
5	150	11715362	11715362
