# Supplementary material for: Microwave Irradiation as a Powerful Tool for Isolating Isoflavones from Soybean Flour
Source: Molecules. 2024 Oct 2;29(19):4685. doi: 10.3390/molecules29194685 (PMC11477798; doi:10.3390/molecules29194685)
Supplement: Supplementary file 1 [file molecules-29-04685-s001.zip › Figure S3.pdf]

Anton Paar GmbH
Anton-Paar-Straße 20
8054 Graz
Austria

Anton Paar Monowave

Serial Number: 81247851
Instrument Software Version: 3.20.7338.3

Processing Protocol

- Experiment Name: sanja_copy3
- Experiment Date: 9/6/2023 1:13:31 PM
- User: Dule

Steps

Step	Program	Temperature	Time	Cooling	Stirrer Speed
		°C	hh:mm:ss		rpm
1	Heat as fast as possible	55	-	On	600
2	Hold	-	00:10:00	On	600
3	Cool down	55	-	On	600

Experiment Result

- Result: OK
- Temperature Control: IR
- User name of last IR sensor adjustment: Administrator
- Vial Type: Glass vial G30

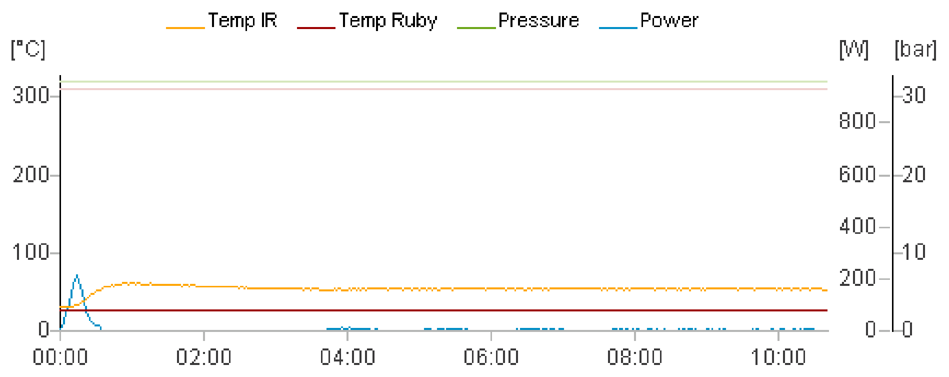

Raw data is accessible for download from the "Browse Results" dialog on Monowave 300 (see serial number above)

Wednesday, September 06, 2023 ______________________________
(signature)

Figure S3. Example of a temperature-controlled mode at 55°C for 10 min, with a graphical representation of the irradiation power change during the experiment
