# Supplementary material for: Microwave Irradiation as a Powerful Tool for Isolating Isoflavones from Soybean Flour
Source: Molecules. 2024 Oct 2;29(19):4685. doi: 10.3390/molecules29194685 (PMC11477798; doi:10.3390/molecules29194685)
Supplement: Supplementary file 1 [file molecules-29-04685-s001.zip › Table S1.pdf]

Table S1. Variation in microwave irradiation power in mode 1 (temperature-controlled mode) and temperature in mode 2 (power-controlled mode) at different extraction times.

**Extraction mode**		**Extraction time**		
		2 min	5 min	10 min
**temperature-controlled mode**	MAE, 55°C	105-0W	153-12W	205-15W
	MAE, 65°C	162-18W	267-20W	315-22W
	MAE, 75°C	230-17W	308-20W	325-23W
	MAE, 85°C	279-20W	350-26W	375-27W
**microwave power-controlled mode**	MAE, 25W	27-65°C	27-78°C	27-96°C
	MAE, 50W	27-86°C	27-95°C	27-106°C
	MAE, 75W	27-96°C	27-106°C	27-118°C
**maceration (24h)**	25°C			
**ultrasonic bath (1h)**	25-37°C			
